# Complete Genome Sequence of Porcine Coronavirus HKU15 Strain IN2847 from the United States

**DOI:** 10.1128/genomeA.00291-14

**Published:** 2014-04-17

**Authors:** Leyi Wang, Yan Zhang, Beverly Byrum

**Affiliations:** Animal Disease Diagnostic Laboratory, Ohio Department of Agriculture, Reynoldsburg, Ohio, USA

## Abstract

Porcine coronavirus HKU15 (PorCoV HKU15) was first detected in pigs with clinical diseases in February 2014 in the United States. Here, we report the complete genome sequence of Indiana strain IN2847, which might be useful for understanding the molecular profile of PorCoV HKU15.

## GENOME ANNOUNCEMENT

Porcine coronavirus HKU15 (PorCoV HKU15), a member of the *Deltacoronavirus* genus of the *Coronaviridae* family, is an enveloped, positive-sense, single-strand RNA virus (1). Previously, only one surveillance study reported detection of PorCoV HKU15 in pigs in Hong Kong (1). It has never been associated with clinical diseases in pigs. On 7 February 2014, a deltacoronavirus (porcine coronavirus HKU15-OH1987) was detected in fecal and small intestine samples from a pig farm in Ohio (unpublished data). Subsequently, the whole genome of the coronavirus was sequenced and the virus was determined to be PorCoV HKU15 (L. Wang, B. Byrum, and Y. Zhang, submitted for publication). Here, we report the whole genome sequence of another PorCoV HKU15 strain, IN2847, which has been detected in Indiana.

Three fecal samples and an intestine sample from pigs experiencing an outbreak of a diarrheal disease at a farm located in Indiana were submitted to the Animal Disease Diagnostic Laboratory, Ohio Department of Agriculture. RNA samples were extracted and tested for porcine epidemic diarrhea virus (PEDV) and PorCoV HKU15. All samples were positive for PorCoV HKU15 by a real-time reverse transcription (RT)-PCR and negative for PEDV. The complete genome of PorCoV HKU15-IN2847 was sequenced and analyzed to determine the genetic relationship of PorCoV HKU15-IN2847, PorCoV HKU15-OH1987, and two strains (PorCoV HKU15-44 and PorCoV KU15-155) in GenBank.

The whole genome sequence of PorCoV HKU15-IN2847 was determined using 16 pairs of overlapping primers designed using two PorCoV HKU15 sequences (strains 44 and 155) in GenBank. The complete genome sequence of PorCoV HKU15-IN2847 is 25,422 nucleotides (nt) in length, excluding the 3′-poly(A) tail. The genome arrangement and corresponding nucleotide positions are as follows: 5′-untranslated region (UTR) (nt 1 to 539), replicase gene (nt 540 to 11,414 for 1a and nt 11,414 to 19,342 for 1b), spike (S) gene (nt 19,324 to 22,806), envelope (E) gene (nt 22,800 to 23,051), membrane (M) gene (nt 23,044 to 23,697), nonstructural 6 (NS6) gene (nt 23,697 to 23,981), nucleocapsid (N) gene (nt 24,002 to 25,030), nonstructural 7 (NS7) gene (nt 24,096 to 24,698), and 3′-untranslated region (nt 25,031 to 25,422).

The complete genome of PorCoV HKU15-IN2847 has a nucleotide identity of 99.2% with HKU15-155 (GenBank accession no. JQ065043), 98.9% with HKU15-44 (GenBank accession no. JQ065042), and 99.9% with HKU15-OH1987 (GenBank accession no. KJ462462). The complete genome comparison showed that HKU15-IN2847 contains two 3-nt insertions (at positions 19,469 and 25,044) in comparison with HKU15-155 and a 1-nt insertion (at position 25,263) in comparison with HKU15-44. In contrast, the two U.S. strains PorCoV HKU15-IN2847 and -OH1987 have the same genome size.

In conclusion, PorCoV HKU15 Indiana strain IN2847 is similar in genetic profile to another U.S. strain of PorCoV HKU15, OH1987, detected in Ohio. The sequence data suggest that both U.S. strains of PorCoV HKU15 were introduced into the United States by a common source. Further study of the virus from other geographic locations in the United States will facilitate our understanding of the genetic diversity and evolution of PorCoV HKU15 in the United States.

### Nucleotide sequence accession number.

The complete genome sequence of PorCoV HKU15 strain IN2847 was submitted to GenBank under the accession no. KJ569769.

## References

[1] Woo PC Lau SK Lam CS Lau CC Tsang AK Lau JH Bai R Teng JL Tsang CC Wang M Zheng BJ Chan KH Yuen KY 2012 Discovery of seven novel mammalian and avian coronaviruses in the genus *Deltacoronavirus* supports bat coronaviruses as the gene source of alphacoronavirus and betacoronavirus and avian coronaviruses as the gene source of *Gammacoronavirus* and *Deltacoronavirus*. J. Virol. 86:3995–4008. 10.1128/JVI.06540-11 22278237 PMC3302495

